# Neighborhood environments influence emotion and physiological reactivity

**DOI:** 10.1038/s41598-019-45876-8

**Published:** 2019-07-01

**Authors:** Daniel A. Hackman, Stephanie A. Robert, Jascha Grübel, Raphael P. Weibel, Eirini Anagnostou, Christoph Hölscher, Victor R. Schinazi

**Affiliations:** 1 USC Suzanne Dworak-Peck School of Social Work, University of Southern California, 669 W. 34th Street, Los Angeles, 90089 California USA; 2 School of Social Work, University of Wisconsin-Madison, 1350 University Ave, Madison, 53706 Wisconsin USA; 3 Chair of Cognitive Science, ETH Zürich, Clausiusstrasse 59/RZ E 23, 8092 Zürich, Zürich, Switzerland

**Keywords:** Emotion, Stress and resilience, Human behaviour, Risk factors

## Abstract

Living in a disadvantaged neighborhood is associated with worse health and early mortality. Although many mechanisms may partially account for this effect, disadvantaged neighborhood environments are hypothesized to elicit stress and emotional responses that accumulate over time and influence physical and mental health. However, evidence for neighborhood effects on stress and emotion is limited due to methodological challenges. In order to address this question, we developed a virtual reality experimental model of neighborhood disadvantage and affluence and examined the effects of simulated neighborhoods on immediate stress and emotion. Exposure to neighborhood disadvantage resulted in greater negative emotion, less positive emotion, and more compassion, compared to exposure to affluence. However, the effect of virtual neighborhood environments on blood pressure and electrodermal reactivity depended on parental education. Participants from families with lower education exhibited greater reactivity to the disadvantaged neighborhood, while those from families with higher education exhibited greater reactivity to the affluent neighborhood. These results demonstrate that simulated neighborhood environments can elicit immediate stress reactivity and emotion, but the nature of physiological effects depends on sensitization to prior experience.

## Introduction

The neighborhoods we live in are important for our health. Independent of individual or family-level characteristics, neighborhood socioeconomic disadvantage is associated with early mortality and worse physical and mental health^1–5^. Understanding the mechanisms underlying these associations is critical for improving population health and for testing theories concerning how broad social and contextual factors influence psychological and biological functioning. Neighborhoods may affect health over the life course through a variety of pathways including social services, health behaviors, physical or material exposures and social factors such as social support, social capital, and social disorder^3–7^.

Stress is also a central mechanism by which neighborhoods are theorized to influence health. Perceiving neighborhood contexts as stressful may induce negative emotion and biological stress responses^3–5,7,8^, which have widespread systemic effects that are thought to link adversity to health and mental health over time^8–12^. Indeed, neighborhood disadvantage is associated with indicators of chronic physiological stress, such as allostatic load^9,13,14^ and the baseline function and diurnal pattern of the hypothalamic-pituitary adrenal (HPA) axis^15,16^. In addition, neighborhood conditions are associated with cortisol and blood pressure reactivity to stressful conditions, though the direction of effects varies and may depend on other factors, such as sex and race/ethnicity^17–20^. Neighborhood environments may not only affect acute stress and emotion, but also may affect chronic stress and emotional functioning due to accumulation of acute stress responses and the long-term toll of adapting to adverse neighborhood environments^6,8,10,11^. Over time, this adaptation may take the form of either habituation (i.e. reduced reactivity to neighborhood conditions over time) or sensitization (i.e. increased reactivity with repeated exposures)^10,21,22^, which have different implications for the processes linking neighborhood stressors and health^8,10,11,22^.

Despite the theoretical importance of stress mechanisms, to our knowledge there are no studies demonstrating that neighborhood environments themselves elicit acute emotional and stress responses, nor are there studies that are able to examine whether neighborhood conditions are associated with patterns of habituation or sensitization. In part, this may be due to methodological challenges. First, there are no standardized neighborhood-related stress response protocols, and thus most studies use stress reactivity tasks in the laboratory that are not related to neighborhood environments or experiences. Second, studies measuring stress and emotion in real neighborhood contexts cannot standardize and control exposures so that comparisons are equivalent across participants.

To address these issues, we developed a novel, virtual reality (VR) experimental model that approximates the experience of being in either a disadvantaged or affluent neighborhood. VR has been used to study highly stressful combat-related exposures^23^, stress reactivity^24–27^, social environment characteristics^28–30^, and habituation in exposure therapy^31^. As such, VR may also be suitable to test the hypothesis that exposure to simulated neighborhood conditions elicits immediate and differential emotional and stress responses. Moreover, acute VR exposures can be utilized in order to test hypotheses concerning habituation and sensitization. Here, prior stressful experiences may influence the response to VR neighborhood conditions, resulting in decreased responses consistent with habituation or increased responses consistent with sensitization.

**Figure 1:**
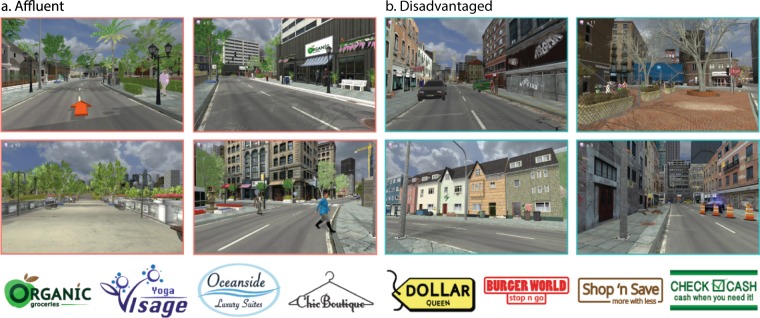
Virtual neighborhoods. Example scenes from virtual (**a**) affluent and (**b**) disadvantaged neighborhoods, along with a sample of the signs created for businesses and services in the neighborhoods. Note: Business names and logos are fictional creations and any correspondence with real businesses is unintentional and coincidental.

We designed two VR neighborhoods to represent disadvantage and affluence (see Fig. 1 and Supplemental Videos S1, S2, showing sample blocks from each neighborhood), using the Unity3D game engine (https://unity3d.com) and the Experiments in Virtual Environments (EVE) computing framework^32^. Neighborhoods were differentiated by building and business types, signals of social and physical disorder (e.g., graffiti, litter), the level of upkeep or deterioration, green space, the presence of amenities and services (e.g., health services, community centers, parks), and other features such as security elements^33–35^. To represent different business types across neighborhoods, signs for business and services were designed and strategically placed on shopfronts and other buildings, such as those for grocery stores, restaurants, fast food, convenience stores, financial services, health care, real estate, lodging, clothing stores, and public services. Attention was also paid to the interaction between features in the neighborhoods, such as how the meaning of a park may change based on whether it is deteriorated or if there are signals such as broken liquor bottles^36^. The neighborhoods were populated by a set of human avatars that reflect the overall demographics of the United States (based on U.S. Census Bureau, Profile of General Population and Housing Characteristics: 2010), balanced to ensure that avatar type (e.g. professional and casual) was not biased by race/ethnicity or gender, and held constant across the two neighborhoods. In addition, each neighborhood was equipped with spatialized background city noise depicting traffic, construction, birds and human vocalizations. Sounds were equivalent across conditions (e.g. traffic, birds, water, human vocalizations), but the amount of each sound changed to remain congruent with the environment (e.g. more sounds of birds and water in well-kept parks).

In the present study, we employed a systematic social observation to verify if virtual neighborhoods were appraised as distinct representations of disadvantage and affluence. In addition, after navigating the neighborhoods, participants reported the emotions they felt while immersed in the neighborhood environment to determine if exposure to disadvantage elicited a more negative affective response than exposure to affluence. To determine whether disadvantaged neighborhood conditions elicited differential autonomic nervous system and cardiovascular stress responses, we measured systolic (SBP) and diastolic (DBP) blood pressure, non-specific skin conductance responses (NS.SCRs), and respiratory sinus arrhythmia (RSA) throughout exposure to different neighborhood conditions. Finally, we also tested the hypothesis that responses depend on prior experience. In particular, we sought to determine if prior stressful experiences, as indexed with parental education^37,38^, were associated with patterns of reactivity to neighborhood conditions that are consistent with habituation or sensitization. In particular, habituation would be evident if participants responded less to experimental neighborhoods that are congruent with their parental education level. In contrast, sensitization would be evident if participants exhibited greater responses to the VR neighborhoods congruent with their parental education level.

## Materials and Methods

### Participants and design

The study was conducted at ETH Zürich, and participants were recruited through the ETH Zürich Decision Science Laboratory online database (DeSciL, https://www.uast.uzh.ch). Participants were eligible for inclusion if they reported they were in good physical and mental health, 18–40 years old, English-speaking, with normal or corrected vision, and able to use their dominant hand for joystick manipulation and non-dominant hand for physiological measurements. Seventy-six participants were recruited, with seven excluded due to technical issues and one due to simulator sickness. Sixty-eight participants were thus randomly assigned to either the affluent or the disadvantaged virtual reality (VR) neighborhood (34 per condition). The majority of participants (98.5%) were graduate or undergraduate students, evenly distributed by sex, with a mean age of 22.7 years (range 18–31). In addition, 64.7 percent of participants had at least one parent with a college degree or higher. Table 1 reports the characteristics of the full sample and each experimental group and illustrates that there were no differences in baseline characteristics between experimental groups (all *p* ≥ 0.17). The study was approved by the ETH Zürich ethics commission (EK 2013-N-73) and performed in accordance with relevant guidelines and regulations. All participants provided written informed consent prior to the start of the experiment.

**Table 1:** Sample descriptive statistics and comparison across experimental conditions.

	Full sample (*n* = 68)	Disadvantaged condition (*n* = 34)	Affluent condition (*n* = 34)	*t*	*p*
	*M (SD)*	*M (SD)*	*M (SD)*		
**Age**	22.7 (2.6)	23.2 (2.8)	22.3 (2.4)	1.38	0.17
**Baseline physiology**					
SBP	105.2 (8.3)	104.0 (8.1)	106.5 (8.4)	1.27	0.21
DBP	65.7 (6.8)	65.7 (7.6)	65.8 (6.1)	0.05	0.96
NS.SCRs (# per minute)	22.0 (7.6)	21.2 (7.5)	22.7 (7.8)	0.81	0.42
RSA	6.2 (1.1)	6.1 (1.1)	6.3 (1.1)	0.84	0.41
**Neighborhood self-report**					
How safe?	2.5 (0.5)	2.5 (0.5)	2.5 (0.5)	0.24	0.81
Overall rating	2.4 (0.6)	2.4 (0.6)	2.5 (0.6)	0.83	0.41
**Simulator sickness**	485.9 (349.8)	550.3 (339.0)	421.5 (353.6)	1.53	0.13
**City navigation time** ** (min)**	20.5 (3.6)	19.1 (3.3)	22.0 (3.4)	3.48	0.001
**Video game hours**	3.3 (5.8)	3.7 (7.1)	2.8 (4.3)	0.65	0.52
	*n* (%)	*n* (%)	*n* (%)	*χ* ^2^	*p*
**Sex**				0.00	1.00
Female	34 (50%)	17 (25%)	17 (25%)		
Male	34 (50%)	17 (25%)	17 (25%)		
**Parental Education**				1.03	0.31
College or Above	44 (64.7%)	24 (35.3%)	20 (29.4%)		
Below College	24 (35.3%)	10 (14.7%)	14 (20.6%)		
**Education Level**				2.33	0.31
High School	1 (1.5%)		1 (2.9%)		
Undergraduate student	40 (58.8%)	18 (52.9%)	22 (64.7%)		
Graduate student	27 (39.7%)	16 (47.1%)	11 (32.4%)		
**Swiss national**	24 (35.3%)	12 (35.3%)	12 (35.3%)	0.00	1.00
**Marital status**				0.73	0.39
Married/Live with partner	6 (8.8%)	2 (5.9%)	4 (11.8%)		
Single	62 (91.2%)	32 (94.1%)	30 (88.2%)		
**Currently smoke or use tobacco**	9 (13.2%)	5 (14.7%)	4 (11.8%)	0.13	0.72

### Neighborhood protocol

**Figure 2:**
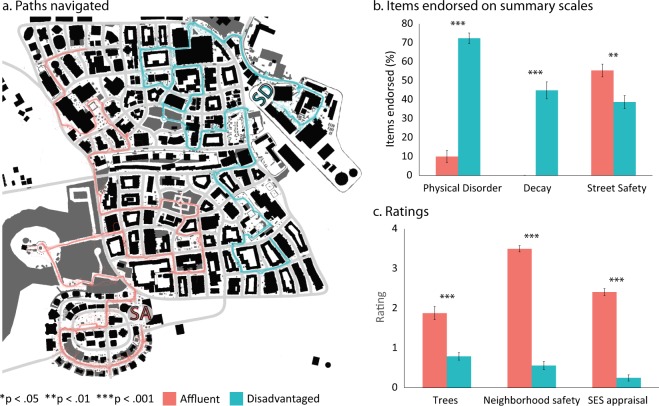
Neighborhood environments: Paths and Perceptions. (**a**) Paths navigated through the neighborhood environments in a virtual city (SA/SD represent the start point for the affluent and disadvantaged routes, respectively). Participants followed the same route with some freedom to explore. Thicker lines represent the most popular paths, while thinner lines represent less common paths some participants explored. (**b**,**c**) Perceptions of neighborhood characteristics for each experimental condition from the SSO ratings^39^. For each SSO measure, bars represent (**b**) mean percentage of items endorsed on each scale, and (**c**) mean ratings, with standard errors. Indices of statistical significance are from the Mann-Whitney U test.

Prior to neighborhood navigation, participants watched a 7-minute nature video of forest and water landscapes to establish a baseline for measurement of physiological reactivity. During neighborhood navigation, participants followed a route marked by intermittent arrows with some limited freedom to explore the environment. Figure 2a shows the paths walked throughout each neighborhood. To prompt visual scanning and promote exploration of the environment, participants were also asked to collect a series of tokens that were strategically placed around the neighborhoods. After neighborhood immersion, participants completed a systematic social observation to quantify and validate their perception of the neighborhood^34,39^. On average it took 20.5 minutes (*SD* = 3.6) to navigate the virtual environment, and there were no differences in the percentage of tokens collected for each neighborhood, *t*(50.99) = 1.77, p = 0.081.

Adult and child avatars were used to enhance the neighborhood social environment. We used Adobe Fuse Character Creator (https://www.adobe.com/products/fuse.html) to create 152 adult avatars, animated with Mixamo’s 3D software (https://www.mixamo.com). Unity’s navigation mesh (NavMesh) feature was employed to define the walkable surfaces, and avatars were spawned from different locations and programmed to walk to a series of destinations. Avatars were also placed at static locations, such as standing on corners or sitting at café tables, with some conversing with each other. The avatars did not interact with participants or respond to attempts at social engagement. In case of collision, the avatars were programmed to apologize with a gesture.

### VR Setup

The VR system included a 55″ ultra-high definition television display (Samsung UE55JU6470, 3840 × 2160 pixels) for desktop presentation and a high-end gaming computer (Dell Alienware Area 51 Base with a 3.8 GHz overclocked i7–5820K processor, dual NVIDIA GeForce GTX 1080 video cards and 32 GB of SDRAM) running Windows 10. Participants were seated facing the television display at a distance of 1.6 meters, used a joystick (Mad Catz V.1 Stick) to navigate, a wireless keyboard and mouse to answer questionnaires, and wore headphones capable of producing spatialized sounds (Logitech G633 Artemis Spectrum with 7.1 Surround).

### Measures

#### Participant characteristics

We used the MacArthur Network Sociodemographic Questionnaire (http://www.macses.ucsf.edu), adapted for use in Switzerland, to collect sociodemographic information. Participants were from 19 different countries across five continents, though the majority were originally from Europe (73.5%).

Participants also rated the quality and safety of their current neighborhood^40^. In terms of overall neighborhood ratings, 48.5% participants rated their neighborhood as “excellent”, 47.1% rated it as “good”, and only 4.4% of participants rated their neighborhood as “fair”. With respect to safety, 51.5% of participants rated their neighborhood as “extremely safe”, while the other 48.5% rated it as “quite safe”.

#### Parental educational level

The maximum level of education achieved by either parent was used as a socioeconomic indicator of prior stressful experiences^37,38^. Parental education was chosen as it is more stable and more likely to be known by participants compared to other socioeconomic indicators from childhood such as income. College degree was chosen (1 = college degree or above, 0 = below a college degree), *a priori*, as it is an important gateway to knowledge, social resources and prestige, and thus is a good retrospective marker of family socioeconomic status (SES).

#### Systematic social observation (SSO)

Participants completed a modified version of the SSO i-Tour^34,39^ to measure appraisal of the neighborhoods. The SSO i-Tour is designed for use in a digital environment (Google Maps/Street View) and generates multiple scales^34^. The *physical disorder* scale is a count of features such as graffiti, garbage or litter on street (range 0–5, 5 = 100% of items endorsed). The *physical decay* scale is a count of the ratings of poor or badly deteriorated conditions across streets, sidewalks, etc. (range 0–4). A *street safety* scale sums reports of the presence of safety measures, such as crosswalks, speed limit signs, and bike lanes (range 0–6). *Neighborhood dangerousness/safety* is the average rating of how safe the neighborhood was overall and how safe they would feel walking there at night (range 0–4). Green space was indicated by estimates of how much of the neighborhood had trees (3 = 75% or more; 2 = 50–74%, 1 = 1–49%, and 0 = None). Finally, participants appraised the neighborhood’s SES, whether it was best characterized as: wealthy/prosperous, comfortably off, of moderate means, or poor. Descriptive results are presented in Fig. 2b,c, and Supplementary Table S1. There were no differences in participant ratings of the weather *χ*2 (2, *N* = 68) = 0.90, *p* = 0.64, or the time of day *χ*2(1, *N* = 68) = 1.34, *p* = 0.25, which were held constant across neighborhoods, suggesting that differences in neighborhood perceptions are unlikely to be due to general differences in reporting.

#### Gaming experience and simulator sickness

To rule out differences in response to VR associated with gaming experience, total hours of weekly video game usage were assessed with a video game playing questionnaire^41^. In addition, the total severity score from the Simulator Sickness Questionnaire^42^ was used to control for differences in emotional and physiological responses that could be due to the VR protocol.

#### Emotional responses

After completing the experiment, participants were asked how they felt while in the neighborhood. The Self-Assessment Manikin (SAM)^43^ measured affect (from happy to unhappy) and alertness/arousal (from excited – calm) on a dimensional scale. In addition, participants reported how strongly they felt nine emotions (amusement, anger, contentment/happiness, compassion, disgust, enthusiasm/excitement, fear, sadness, and surprise, 0–8 scale)^44^. Supplementary Table S2 presents the descriptive statistics and correlations among responses. Composite measures were created for positive (amusement, contentment/happiness, enthusiasm/excitement) and negative emotions (anger, disgust, fear, sadness) by averaging the z-scores for individual emotions. Analyses were repeated utilizing two factors, corresponding to positive and negative emotion, using both promax and varimax rotations. Findings follow the same pattern as those reported using composites (analyses available upon request). Compassion and surprise were examined individually in exploratory analyses because they positively correlated with individual measures of both positive and negative emotions.

#### Physiological reactivity

Blood pressure, electrodermal activity and respiratory sinus arrhythmia (RSA) were utilized as indices of autonomic nervous system and cardiovascular stress responses^45–47^.

**Blood pressure:** Systolic (SBP) and diastolic (DBP) blood pressure were recorded at 3-minute intervals (Phillips SureSigns VS2 + ). One participant was omitted from analyses for DBP as multiple readings were greater than 3 SD above the mean. Three measures of SBP were not recorded by the system. Consequently SBP analyses included 462 measures for 68 participants and DBP analyses included 458 observations for 67 participants.

**Electrodermal activity (EDA):** EDA was collected with two finger electrodes (MLT118F, ADInstruments) attached to the middle segment of the index and ring finger. Electrodes were connected to the PowerLab 8/35 recording device through a FE116 GSRAmp signal amplifier (ADInstruments). Data were recorded at a sample rate of 1000 Hz using LabChart version 8.1.4 (ADInstruments). Temperature (*M* = 22.8 °C (range 21.5–24) and humidity (*M* = 35.9%, range 31–51) in the testing room were kept within the optimal range^48,49^, with no differences by experimental condition for temperature, *t*(66) = −1.13, p = 0.26, or humidity, *t*(66) = 0.98, p = 0.33.

Given the duration of neighborhood exposure and the multiplicity of potentially evocative stimuli, it was hypothesized that neighborhood exposure would result in heightened reactivity overall, or an increased number of responses to an unpredictable set of stimuli. Consequently, analyses focused on non-specific skin conductance responses (NS.SCR’s). NS.SCR’s are discrete increases in skin conductance that are not linked to a specific stimulus^48^ and defined at a threshold 0.01 µS for each response window. Total NS.SCR’s were calculated per minute, with 1,361 observations across participants.

Matlab (R2017a, MathWorks, Natick, MA, USA) was used to export events from LabChart and to separate them into 1-minute interval windows. Data were eliminated for intervals that were less than one-minute. Data were processed using Ledalab software version 3.4.9 (www.ledalab.de), and were first down sampled to 10 Hz and visually inspected for artifacts, none of which were found. We used continuous decomposition analysis (CDA)^50^ to extract NS.SCR’s. Values were optimized two times to improve the estimation of the parameters of the impulse response function.

**Respiratory sinus arrhythmia (RSA):** Electrocardiography (ECG) was collected using the ADInstruments Powerlab 8/35 Data Acquisition System with a Bio Amp (FE132, ADInstruments) and three disposable electrodes (MLA1010, ADInstruments) placed on the second intercostal space below the middle of the clavicle on the right (RA) and left (LA) side of the chest accordingly. A third electrode was attached below the 9th left rib (LL)^51^. ECG was sampled at a rate of 1000 Hz, with LabChart data acquisition software (LabChart v8.1.4, ADInstruments). ECG data were analyzed with Kubios HRV software, version 3.1^52^. The time series of interbeat intervals was generated for each participant and visually inspected for correct beat identification. Kubios also automatically identified and corrected artifacts – these corrections were visually inspected and impacted 0.71% of overall data points and no more than 3.12% of beats for any individual participant. The signal was processed using the very low threshold filter setting (0.45 sec) and the smooth priors filter for detrending (λ = 500, fc = 0.035 Hz). Data were analyzed in one-minute epochs, using a Fast Fourier transform focusing on power in the high frequency range (0.15–0.4 Hz) reflective of parasympathetic nervous system activity^53,54^. Data were eliminated for epochs that were less than one-minute or for which ECG derived respiration estimates indicated that respiration frequency was out of the high frequency band. Absolute values of high frequency power were natural log transformed, generating a measure of RSA for each minute of the protocol. The RSA analysis thus consists of 1,325 observations from 67 participants across the protocol.

### Procedures

The experiment was conducted in a curtained, private area at the NeuroLab at ETH Zürich. Participants were asked to abstain from caffeine, tobacco, alcohol and exercise for at least three hours before the experiment. Participants used a joystick to navigate, which they were trained to use, and practiced in a virtual maze to ensure they were adept with the control interface and task procedures and had time to recover from any initial, general response to the VR environment (see Supplementary Fig. S1 for full experimental protocol). The experiment was deployed within EVE (Experiments in Virtual Environments)^32,51^, a comprehensive computing framework for the presentation of virtual environments and the collection of behavioral and physiological data (available at https://cog-ethz.github.io/EVE/).

### Data analysis

To determine if there were differences in perception of the affluent and disadvantaged neighborhoods, we utilized the Mann-Whitney U test, given violations of assumptions for normal distribution (Fig. 2b,c, and Supplementary Table S1). These analyses, and all regression models, were conducted using IBM SPSS versions 24 and 25. All statistical tests were two-sided.

We examined the main effect of neighborhood condition on emotion and physiological responses to determine if there was an acute effect. Moderation analyses were used to test the hypothesis that responses depend on prior experience, and if they are consistent with habituation or sensitization, by examining the interaction between parental education and VR neighborhood condition. We employed multiple linear regression models for analyses of emotional responses.

For physiological measures, we used linear mixed-effects modeling in SAS 9.4 to account for repeated measures. Models were estimated using restricted maximum likelihood and an unstructured covariance structure. To determine the best fitting model, we started with an unconditional model and then added fixed and random effects for linear and quadratic terms for time during the experiment, comparing models utilizing-2 Log-likelihood and the Akaike and Bayes Information Criterion (AIC/BIC). For models of SBP and DBP, the best fitting model included both a random intercept and fixed effect for a linear index of time, in minutes, during the experiment. For NS.SCRs and RSA, the best fitting and most parsimonious model included a random intercept and both a random and fixed effect for the linear index of time. Due to inconsistency across fit criteria for NS.SCRs, we also examined models that added random and fixed effects for a quadratic index of time. Results were broadly equivalent to models without quadratic terms, and thus we chose the most parsimonious model. Baseline estimates of SBP, DBP, NS.SCR, and RSA correspond to the average of measurements during the 7-minute nature video. This baseline measure was subtracted from each data point during neighborhood exposures such that each repeated measure represents reactivity in comparison to baseline. Consequently main effects and interactions in the model can be interpreted as predicting overall blood pressure, RSA, or NS.SCR reactivity to the task. We also conducted exploratory analyses to examine potential differences in the growth rate of reactivity between experimental conditions and based on parental education. As no interactions with time were significant (all *p* ≥ 0.11), they were not included in models. Effect sizes were calculated using Cohen’s *d*, with the interaction effect size being the difference between the effect size for the experimental condition at each level of parental education.

Finally, we ran models controlling for *a priori* covariates to ensure effects were specific (see Supplementary Tables S3–S8). These models were run individually due to the study sample size, which precludes including all covariates in a single model. Covariates included age, sex, current education level (Graduate Student = 1, Undergraduate or Not in School = 0), hours of video games played, national origin (Swiss National versus other), smoking status, subjective perceptions of current neighborhood safety and quality, simulator sickness, as well as the amount time navigating the neighborhoods, which was longer for the affluent condition, *t*(66) = 3.48, *p* = 0.001).

## Results

Participants perceived the neighborhoods to be distinct and in concert with the type of neighborhood differences observed with systematic social observations^34,39^ (see Fig. 2b,c and Supplementary Table S1). Participants that navigated the disadvantaged neighborhood reported significantly higher levels of physical disorder, Mann-Whitney U-test, *U*(68) = 32, *p* < 0.001, and decay, *U*(68) = 51, *p* < 0.001, compared to those that navigated the affluent neighborhood. Ratings of street safety, *U*(68) = 324.5, *p* = 0.001, appraisal of trees in the neighborhood, *U*(68) = 189.5, *p* < 0.001, and overall appraisal of safety, *U*(68) = 0.0, *p* < 0.001, were rated higher in the affluent neighborhood than in the disadvantaged neighborhood. In addition, the overall perceived socioeconomic status (SES) was rated significantly higher in the affluent neighborhood, *U*(68) = 0.0, *p* < 0.001. Indeed, all participants rated the affluent neighborhood as one of “wealth/prosperity” or “comfortably off”, and the disadvantaged neighborhood as one of “moderate means” or “poor”.

**Figure 3:**
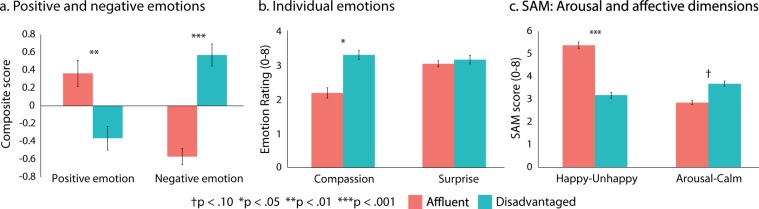
Emotional responses. Panels illustrate emotional responses to different neighborhood conditions for (**a**) composite measures of positive and negative emotion; (**b**) compassion and surprise; and (**c**) dimensional measures of affect and arousal. Bars represent mean composite scores, or mean scores for individual measures, with standard errors. Indices of statistical significance are from linear regression models.

Exposure to the disadvantaged neighborhood, compared to the affluent neighborhood, was associated with higher composite scores of negative emotion, *β* = −0.68, *t*(66) = −7.46, *p* < 0.001, lower composite scores of positive emotion, *β* = 0.41, *t*(66) = 3.65, *p* = 0.001, as well as less happiness on a dimensional scale of affect^43^, *β* = 0.55, *t*(66) = 5.38, *p* < 0.001 (see Fig. 3 and Supplementary Tables S3–S5). Moreover, exposure to the disadvantaged neighborhood was associated with greater compassion during the task, *β* = −0.27, *t*(66) = −2.29, *p* = 0.025, (see Supplementary Table S6) though it was not associated with arousal, *β* = −0.21, *t*(66) = −1.77, *p* = 0.082, or surprise, *β* = −0.04, *t*(66) = −0.29, *p* = 0.77 (see Fig. 3). These associations were independent of age, sex, current education level, video game usage, national origin, smoking status, ratings or comparisons of current neighborhood safety and quality, simulator sickness, and time navigating the city (Supplementary Tables S3–S6). However, none of the interactions between neighborhood condition and parental education were significant (all *p* ≥ 0.07; see Supplementary Tables S3–S6) and thus there was no evidence of either habituation or sensitization of emotional responses to neighborhood conditions.

Mixed effects models accounting for repeated measures indicated that, compared to baseline, there were no main effects of neighborhood condition for overall systolic blood pressure (SBP) reactivity, *B* = −0.04, *t*(393) = −0.06, *p* = 0.95, *d* = −0.002, diastolic blood pressure (DBP) reactivity, *B* = 0.38, *t*(390) = 0.57, *p* = 0.57 *d* = −0.13, electrodermal reactivity [non-specific skin conductance responses (NS.SCR)], *B* = −0.27*, t*(1225) = −0.15, *p* = 0.88, *d* = 0.10, or respiratory sinus arrhythmia (RSA) reactivity, *B* = −0.01*, t*(1191) = −0.08, *p* = 0.93, *d* = 0.05. However, there was an interaction between VR condition and parental education for SBP reactivity, *B* = −2.98*, t*(393) = −2.04*, p* = 0.043, *d* = 0.76, and electrodermal (NS.SCR) reactivity, *B* = −8.44*, t*(1225) = −2.28, *p* = 0.023, *d* = 0.61, indicative of moderation, but not for DBP reactivity, *B* = −1.69*, t*(390) = −1.18, *p* = 0.24, *d* = 0.39, or for RSA reactivity, *B* = −0.01*, t*(1191) = −0.03, *p* = 0.97, *d* = 0.007. For SBP this interaction remains significant when controlling for all covariates except for current education level and current neighborhood quality in comparison to others in the area (Supplementary Table S7). For NS.SCRs, this interaction remained significant when controlling for all covariates (Supplementary Table S8). Consequently, there is evidence that SBP and electrodermal reactivity to neighborhood conditions depend on parental education level.

**Figure 4:**
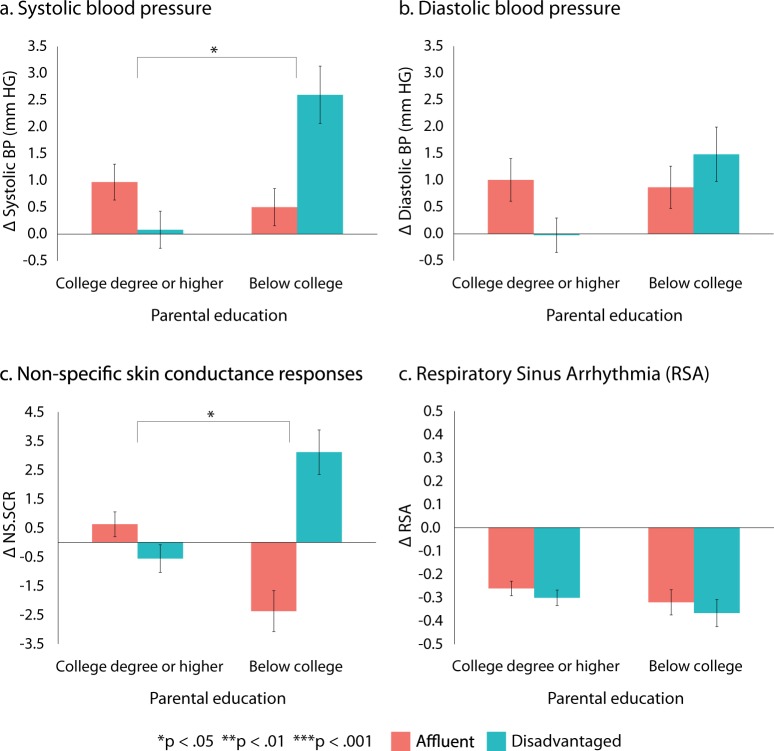
Physiological responses to VR neighborhoods by parental education level. Bars represent mean change compared to baseline, based on the raw data, for all repeated measures across the entire experiment (with standard errors). Indices of statistical significance are for the interaction between experimental condition and parental education, from mixed model analyses.

As illustrated in Fig. 4, this interaction indicates that those whose parents had less than a college degree exhibited greater SBP reactivity (*d* = 0.55) and increases in NS.SCRs (*d* = 0.49) to the disadvantaged neighborhood, while those whose parents had a college degree or above were more reactive to the affluent neighborhood (SBP, *d* = −0.21; NS.SCRs, *d* = −0.12). This pattern is consistent with sensitization, rather than habituation, as there was increased reactivity for participants in the neighborhood condition that was congruent with their parental SES.

## Discussion

This study provides the first support for the hypothesis that acute exposure to different neighborhood environments elicits differences in stress and emotional reactivity. Participant observations of the virtual environments indicated that they were perceived distinctly and as characterizing a more affluent versus disadvantaged neighborhood^33–35^. These results suggest that the virtual neighborhoods are a valid experimental model for use in studying the influence of neighborhood environments in a lab environment.

With respect to emotion, neighborhood disadvantage elicited more negative emotion and less positive emotion, with no evidence of habituation or sensitization. Overall, this is consistent with the role of emotion as a mediator of neighborhood effects on health and mental health, especially for neighborhood differences in mood disorder symptoms^1,3–5,7^. In particular, it suggests that acute exposure to more disadvantaged neighborhoods is associated with positive and negative emotion and, over time, these experiences may add up to influence mood and intersect with other life stressors. In addition to contributing to mood disorders, subclinical negative and positive emotional effects may contribute to differences in physical health via physiological or behavioral mechanisms^55–57^. However, such interpretations require caution and replication across additional, diverse samples. Moreover, the fact that exposure to disadvantage also elicited greater compassion suggests that these effects may be far more complex, and neighborhood exposures may also have potentially positive consequences.

In contrast, we did not find that neighborhood conditions elicit physiological stress or emotional responses by themselves, but instead that patterns of responding were dependent on prior experience. In particular, lower parental education was associated with greater reactivity to disadvantaged neighborhoods while higher parental education was associated with greater reactivity to affluent neighborhoods, though the effect in the affluent neighborhood was smaller in magnitude. These results are consistent with a pattern of sensitization to prior experience, in which continued exposure to stressors do not lead to habituation but leads instead to greater reactivity^10,11,22^. These findings also suggest that greater frequency or amplitude of stress responses may be part of the link between neighborhood stressors and health^10^, and are consistent with studies that have indicated that neighborhood disadvantage is associated with greater allostatic load^9,13,14^. Consequently, there is some evidence that, in a manner dependent on prior experience, neighborhood environments can elicit stress responses that may potentially contribute to differences in health.

In addition, we found different patterns across measures of physiological reactivity and, in particular, evidence of sensitization processes for blood pressure and electrodermal activity but not for respiratory sinus arrhythmia (RSA). RSA is a marker of parasympathetic nervous system activity and has been conceptualized as a peripheral biomarker of emotion regulation and the regulation of cardiac responses to environmental signals^58–60^. This raises the possibility that sensitization processes are specific to stress and arousal but do not encompass processes related to the regulation of emotional responses to neighborhood conditions. Additional studies are needed to confirm such speculation, and must include other measures such as HPA-axis reactivity, to determine the degree to which neighborhood influences on stress reactivity and sensitization-related processes are system-specific or general in nature.

Previous research has already shown that VR protocols can be successful in eliciting social stress. Studies utilizing a VR adaptation the Trier Social Stress Test^24–27^ have revealed that VR-based social stress can elicit systematic differences in physiological reactivity, some of which are comparable to the same stressor *in vivo*. Similarly, a VR protocol has been used to examine the effect of social stressors in a virtual bar, conceptualized as differences in avatar density, hostile facial expressions, and their degree of congruence with the race/ethnicity of the participant^28–30^. In case-control studies of psychosis liability, variation in these stressors have been associated with subjective distress, both for main effects^30^ and as moderated by prior trauma,^29^ and a small pilot study found some differences related to skin conductance^28^. Although the sample and stressor type differ, these studies also converge to suggest that VR can be feasibly deployed to study the influence of broader social and contextual environment on stress and emotion. Nonetheless, the current study is the first to examine the variation in broader, macro-level neighborhood environments in VR as a stressor.

Our study also points to the importance of considering which neighborhood features or characteristics (e.g. green space, nature, social and physical disorder) are most important for stress and emotion^3–7^. Some of these features are currently being studied as part of a growing literature that typically reports positive effects of short-term exposure to more natural environments on stress and emotion^61–64^. However, this strand of research has yet to examine the effects of different neighborhood environments or interactions between nature and neighborhood environments. Congruent with our findings, these studies raise the possibility that differences in stress and emotion may, in fact, account for the role of specific neighborhood features such as nature, or disorder, on health. As such, there may be an overlap or intersection between stress and other hypothesized mechanisms underlying neighborhood effects on health. Future studies should aim to systematically decompose complex VR neighborhood environments into constituent features to determine which, if any, are of greatest salience for stress and emotion.

The inferences that can be drawn concerning sensitization and habituation from this study are limited, in part, by the use of parental education as a retrospective index of prior stressful experiences. Parental education, in this study, was used as a reliable retrospective measure of family socioeconomic status (SES) that captures variation in social prestige, resources, and stressful experiences both within the family and in the family’s broader context^37,38,65–67^. Despite its utility, this marker does not assess psychosocial or physiological stress directly nor does it allow for investigation of stressor specificity, or which aspects of experience are most salient for habituation or sensitization. As such, a categorical indicator such as parental education may underestimate the degree to which prior stressful experiences moderate responses to the VR neighborhood environments. Parental education also does not specifically assess both objective and subjective lifecourse neighborhood experiences, which would provide the most specific test of sensitization and habituation processes related to neighborhood experiences themselves. These results, therefore, speak more to sensitization to prior experience more generally, rather than in terms of chronic neighborhood exposures. Future studies should utilize both measures of psychosocial and physiological stressors as well as lifecourse neighborhood experiences in order to more robustly examine potential habituation and sensitization-related processes.

Given the nature of the sample recruited, it is also possible that some of our findings are not generalizable. On the whole, participants were of higher family SES overall, well-educated, and students that currently live in good neighborhoods in an affluent region in Zurich. This range of experiences may influence how the VR neighborhoods are perceived, given that such perceptions are likely anchored in prior experience. It is also possible that the limited diversity of the sample, particularly in terms of SES and neighborhood exposures, may lead to an underestimation of effects compared to a broader, more diverse sample with more extensive experience with a range of neighborhood types. In order to make more generalizable inferences concerning neighborhood influences on stress, emotion and patterns of sensitization and habituation, it is critical that future studies be conducted with more diverse samples in additional geographic regions.

Beyond the generalizability of the current findings, examining consistency or variation of these patterns across different populations is important for testing more nuanced hypotheses concerning the influence of chronic stress and chronic neighborhood exposures, as this interaction suggests that autonomic and cardiovascular reactivity is most illuminating at the intersection between prior experience and acute exposure to neighborhood conditions. Consequently, in addition to studying more diverse samples, it is also important for such future work to examine how patterns of sensitization might emerge longitudinally, and which prior life course exposures, particularly at the neighborhood level, may account for these patterns. Prior experience may create physiological changes that drive these sensitization processes, or it may be that prior experience alters both the meaning or appraisal of potential stressors or promotes increased knowledge of and vigilance to the salient challenges in one’s typical environment. Only studies with more diverse samples with more complete characterization of lifecourse processes will be able to address such issues.

Despite limitations, results of this study support the validity and feasibility of utilizing VR as an experimental model to study some aspects of acute neighborhood influences on stress and emotion and to test hypotheses concerning how such effects differ based on prior experience. Moreover, these results provide support for the theoretical proposition that neighborhood contexts elicit different patterns of emotion and stress that are relevant for disparities in health and development.

## Supplementary information

Supplementary Information

Video S1: Disadvantaged neighborhood

Video S2: Affluent neighborhood
