# Supplementary material for: Neighborhood environments influence emotion and physiological reactivity
Source: Sci Rep. 2019 Jul 1;9:9498. doi: 10.1038/s41598-019-45876-8 (PMC6602955; doi:10.1038/s41598-019-45876-8)
Supplement: Supplementary file 1 — Supplementary Information [file 41598_2019_45876_MOESM1_ESM.pdf]

Supplementary Information

Neighborhood environments influence emotion and physiological reactivity

Authors: Daniel A. Hackman, Stephanie A. Robert, Jascha Grübel, Raphael P. Weibel, Eirini Anagnostou, Christoph Hölscher & Victor R. Schinazi

Corresponding author: Daniel A. Hackman
Email: dhackman@usc.edu

This PDF file includes:

Fig. S1
Tables S1 to S8

Additional Supplementary Information

Video S1. Video of the virtual environment: Sample blocks of the disadvantaged condition
Video S2. Video of the virtual environment: Sample blocks of the affluent condition

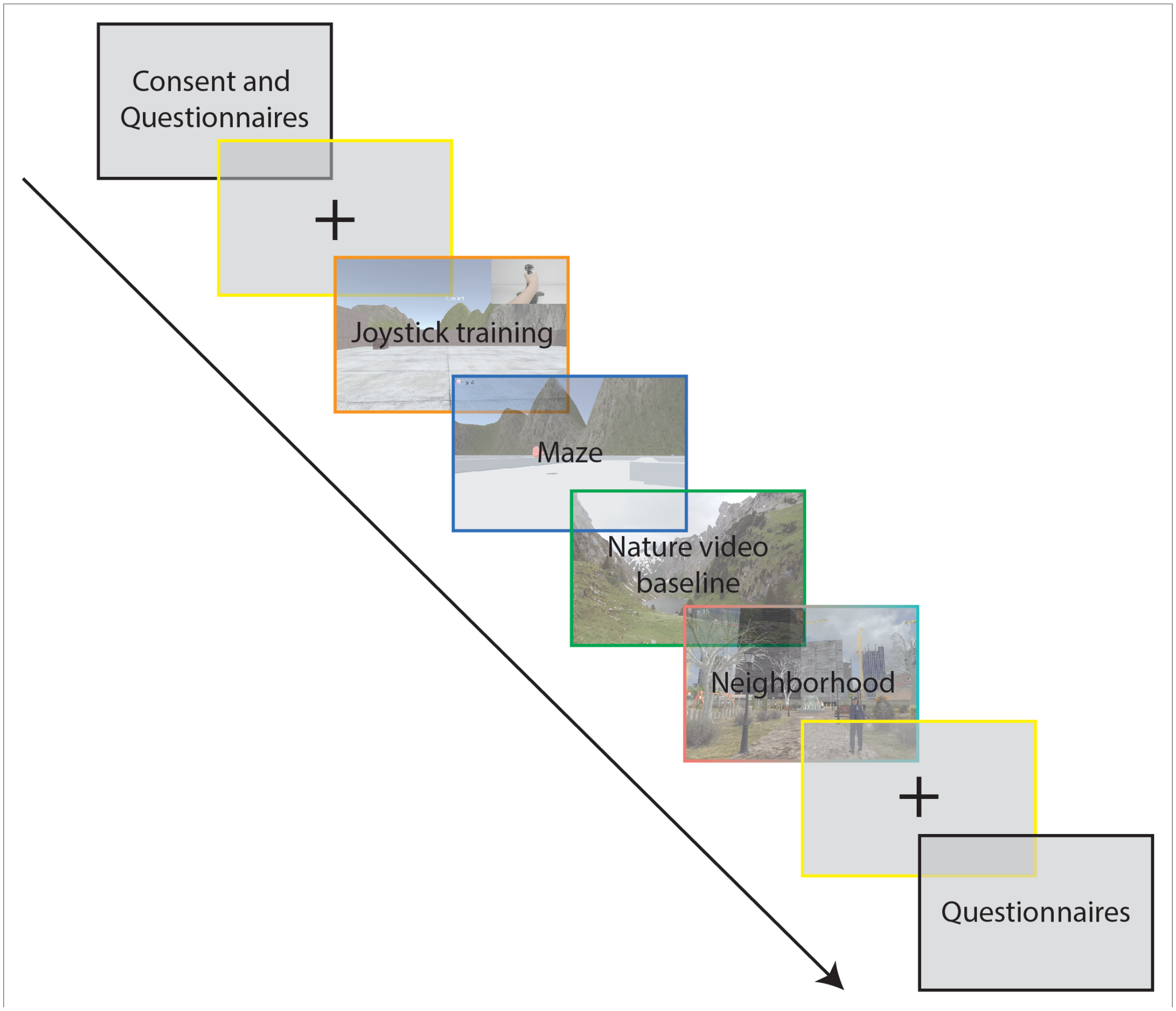

Figure S1. Experimental Procedure
After the informed consent process, participants completed the sociodemographic and video game questionnaires. Subsequently, participants completed an interactive tutorial in order to learn to navigate with the joystick and practice with the joystick inside a virtual maze. After completing the maze, participants watched a nature video to establish baseline for reactivity. At the end of the nature video, participants were randomly assigned to either the affluent or disadvantaged neighborhood for the main task. Before the end of the experiment, participants completed a last round of questionnaires that included a systematic social observation, and self-reports concerning simulator sickness and emotional responses in the neighborhood environments.

Table S1. Full descriptive statistics regarding participant perceptions of the affluent and disadvantaged virtual neighborhoods, as reported using the Systematic Social Observation i-Tour

	Affluent condition (*n* = 34)	Disadvantaged condition (*n* = 34)
	M (SD) or n (%)	*M (SD) or n* (%)
Physical Disorder Scale		
Mean (SD)	0.5 (0.9)	3.6 (0.8)
0 (No items endorsed)	23 (67.6%)	0 (0%)
1	8 (23.5%)	0 (0%)
2	1 (2.9%)	3 (8.8%)
3	1 (2.9%)	11 (32.4%)
4	1 (2.9%)	16 (47.1%)
5 (100% of items endorsed)	0 (0%)	4 (11.8%)
Decay Scale		
Mean (SD)	0.0 (0.0)	1.8 (1.0)
0 (No items endorsed)	34 (100%)	3 (8.8%)
1	0 (0%)	11 (32.4%)
2	0 (0%)	12 (35.3%)
3	0 (0%)	6 (17.6%)
4 (100% of items endorsed)	0 (0%)	2 (5.9%)
Street Safety		
Mean (SD)	3.3 (1.2)	2.3 (1.2)
0 (No items endorsed)	0 (0%)	1 (2.9%)
1	2 (5.9%)	9 (26.5%)
2	7 (20.6%)	9 (26.5%)
3	8 (23.5%)	9 (26.5%)
4	13 (38.2%)	5 (14.7%)
5	3 (8.8%)	1 (2.9%)
6 (100% of items endorsed)	1 (2.9%)	0 (0%)
Neighborhood safety / dangerousness	3.5 (0.5)	0.6 (0.6)
Trees: Percentage presence in neighborhood		
Mean (SD)	1.9 (0.9)	0.8 (0.5)
0 (None)	3 (8.8%)	9 (26.5%)
1 (1 - 49%)	7 (20.6%)	22 (64.7%)
2 (50 - 74%)	13 (38.2%)	2 (5.9%)
3 (≥ 75%)	9 (26.5%)	0 (0%)
Unsure	2 (5.9%)	1 (2.9%)
Neighborhood SES Appraisal		
Mean (SD)	2.4 (0.5)	0.3 (0.4)
0 (Poor)	0 (0%)	24 (70.6%)
1 (Moderate means)	0 (0%)	8 (23.5%)
2 (Comfortably off)	20 (58.8%)	0 (0%)
3 (Wealth / Prosperity)	14 (41.2%)	0 (0%)
Unsure		2 (5.9%)
Weather conditions		
Clear and Sunny	10 (29.4)	6 (17.6%)
Sunny with Clouds	20 (58.8)	18 (52.9%)
Clouds without visible Sun	4 (11.8%)	6 (17.6%)
Unsure	0 (0%)	3 (8.8%)
Time of Day		
Morning	8 (23.5)	13 (38.2%)
Afternoon	23 (67.6)	20 (58.8%)
Unsure	3 (8.8)	1 (2.9%)

Table S2. Correlation table and descriptive statistics of individual emotional responses to VR neighborhoods across all participants.

	M(SD)	1	2	3	4	5	6	7	8	9	10	11
1. Amusement	3.4 (1.9)	--										
2. Anger	1.7 (2.0)	-.17	--									
3. Contentment / Happiness	2.9 (2.0)	.64***	-.28*	--								
4. Compassion	2.7 (2.1)	.26*	.27*	.16	--							
5. Disgust	2.8 (2.6)	-.25*	.49***	-.52***	.41***	--						
6. Enthusiasm / Excitement	2.8 (1.7)	.82***	-.032	.64***	.42***	-.17	--					
7. Fear	1.8 (2.1)	-.14	.53***	-.45***	.28*	.69***	-.11	--				
8. Sadness	2.5 (2.6)	-.25*	.53***	-.54***	.38**	.86***	-.16	.68***	--			
9. Surprise	3.1 (1.6)	.42***	.20	.23†	.33**	.18	.34**	.23†	.15	--		
10. SAM – Happy / Unhappy	4.3 (2.0)	.60***	-.42***	.66***	-.10	-.62***	.52***	-.65***	-.55***	.11	--	
11. SAM – Excited / Calm	3.3 (2.0)	.04	.27*	-.10	.17	.27*	.20	.51***	.23†	.31**	-.29*	--

† $p < .10$, * $p < .05$, ** $p < .01$, *** $p < .001$

Table S3. Negative emotion: Response to virtual neighborhoods

	Model 1		Model 2		Model 3		Model 4		Model 5		Model 6		Model 7		Model 8		Model 9		Model 10		Model 11		Model 12		Model 13		Model 14	
	β	*p*	*β*	*p*	*β*	*p*	*β*	*p*	*β*	*p*	*β*	*p*	*β*	*p*	*β*	*p*	*β*	*p*	*β*	*p*	*β*	*p*	*β*	*p*	*β*	*p*	*β*	*p*
Condition (Affluent)	-0.68	<.001	-0.69	<.001	-0.68	<.001	-0.69	<.001	-0.68	<.001	-0.68	<.001	-0.67	<.001	-0.68	<.001	-0.67	<.001	-0.68	<.001	-0.68	<.001	-0.67	<.001	-0.65	<.001	-0.73	<.001
Parental Education (Below College)			.08	.42	0.08	.57																						
Condition x Parental Education					-0.004	.98																						
Age							-0.06	.49																				
Sex (Female = 1)									0.08	.38																		
Education: Graduate student											-0.05	.61																
Video game hours													0.07	.43														
Swiss National															-0.01	.94												
Smoking																	0.16	.074										
Neighborhood Safety																			-0.08	.40								
Neighborhood Rating																					0.01	.88						
Neighborhood Comparison																							0.01	.29				
Simulator Sickness																									0.15	.12		
City Time																											0.13	.19

Table reports regression models predicting negative emotion

- Model 1 examines the main effect of neighborhood condition with no covariates:
- Models 2 and 3 examine the effect of adding parental education as a covariate (Model 2) and its interaction with condition, to test hypotheses concerning sensitization and habituation (Model 3)
- Models 4 – 14 examine the robustness of the main effect of condition by sequentially testing independence from additional covariates

Table S4. Positive emotion: Response to virtual neighborhoods

	Model 1		Model 2		Model 3		Model 4		Model 5		Model 6		Model 7		Model 8		Model 9		Model 10		Model 11		Model 12		Model 13		Model 14	
	β	*p*	*β*	*p*	*β*	*p*	*β*	*p*	*β*	*p*	*β*	*p*	*β*	*p*	*β*	*p*	*β*	*p*	*β*	*p*	*β*	*p*	*β*	*p*	*β*	*p*	*β*	*p*
Condition (Affluent)	0.41	.001	0.38	.001	0.35	.014	0.44	<.001	0.41	.001	0.43	<.001	0.42	<.001	0.41	.001	0.41	.001	0.41	.001	0.43	<.001	0.41	.001	0.38	.001	0.45	<.001
Parental Education (Below College)			0.25	.027	0.20	.22																						
Condition x Parental Education					0.08	.69																						
Age							0.19	.10																				
Sex (Female = 1)									.03	.81																		
Education: Graduate student											0.11	.34																
Video game hours													0.16	.15														
Swiss National															0.05	.41												
Smoking																	-0.03	.78										
Neighborhood Safety																			-0.10	.40								
Neighborhood Rating																					-0.24	.033						
Neighborhood Comparison																							-0.04	.70				
Simulator Sickness																									-0.14	.21		
City Time																											-0.10	.42

Table reports regression models predicting positive emotion:

- Model 1 examines the main effect of neighborhood condition with no covariates
- Models 2 and 3 examine the effect of adding parental education as a covariate (Model 2) and its interaction with condition, to test hypotheses concerning sensitization and habituation (Model 3)
- Models 4 – 14 examine the robustness of the main effect of condition by sequentially testing independence from additional covariates

Table S5. Positive / Negative Affect (SAM): Response to virtual neighborhoods

	Model 1		Model 2		Model 3		Model 4		Model 5		Model 6		Model 7		Model 8		Model 9		Model 10		Model 11		Model 12		Model 13		Model 14	
	β	*p*	*β*	*p*	*β*	*p*	*β*	*p*	*β*	*p*	*β*	*p*	*β*	*p*	*β*	*p*	*β*	*p*	*β*	*p*	*β*	*p*	*β*	*p*	*β*	*p*	*β*	*p*
Condition (Affluent)	0.55	<.001	0.53	<.001	0.66	<.001	0.58	<.001	0.55	<.001	0.55	<.001	0.55	<.001	0.55	<.001	0.55	<.001	0.55	<.001	0.56	<.001	0.55	<.001	0.50	<.001	0.64	<.001
Parental Education (Below College)			0.22	.035	0.41	.006																						
Condition x Parental Education					-0.31	.069																						
Age							0.14	.19																				
Sex (Female = 1)									-0.13	.23																		
Education: Graduate student											0.02	.89																
Video game hours													-0.00	.97														
Swiss National															0.02	.85												
Smoking																	-0.12	.26										
Neighborhood Safety																			-0.13	.22								
Neighborhood Rating																					-0.11	.28						
Neighborhood Comparison																							-0.05	.64				
Simulator Sickness																									-0.27	.010		
City Time																											-0.21	.056

Table reports regression models predicting positive / negative affect using the SAM (Happy-Unhappy):

- Model 1 examines the main effect of neighborhood condition with no covariates
- Models 2 and 3 examine the effect of adding parental education as a covariate (Model 2) and its interaction with condition, to test hypotheses concerning sensitization and habituation (Model 3)
- Models 4 – 14 examine the robustness of the main effect of condition by sequentially testing independence from additional covariates

Table S6. Compassion: Response to virtual neighborhoods

	Model 1		Model 2		Model 3		Model 4		Model 5		Model 6		Model 7		Model 8		Model 9		Model 10		Model 11		Model 12		Model 13		Model 14	
	β	*p*	*β*	*p*	*β*	*p*	*β*	*p*	*β*	*p*	*β*	*p*	*β*	*p*	*β*	*p*	*β*	*p*	*β*	*p*	*β*	*p*	*β*	*p*	*β*	*p*	*β*	*p*
Condition (Affluent)	-0.27	.025	-0.30	.014	-0.39	.009	-0.28	.023	-0.27	.026	-0.28	.026	-0.25	.033	-0.27	.025	-0.26	.027	-0.28	.023	-0.24	.039	-0.28	.023	-.27	.028	-0.31	.019
Parental Education (Below College)			0.21	.085	0.07	.70																						
Condition x Parental Education					0.22	.28																						
Age							-0.06	.61																				
Sex (Female = 1)									0.09	.47																		
Education: Graduate student											-0.03	.84																
Video game hours													0.21	.072														
Swiss National															0.15	.20												
Smoking																	0.27	.021										
Neighborhood Safety																			-0.13	.27								
Neighborhood Rating																					-0.35	.003						
Neighborhood Comparison																							-0.13	.29				
Simulator Sickness																									-0.01	.94		
City Time																											0.10	.45

Table reports regression models predicting compassion:
- Model 1 examines the main effect of neighborhood condition with no covariates
- Models 2 and 3 examine the effect of adding parental education as a covariate (Model 2) and its interaction with condition, to test hypotheses concerning sensitization and habituation (Model 3)
- Models 4 – 14 examine the robustness of the main effect of condition by sequentially testing independence from additional covariates

Table S7. Systolic Blood Pressure: Response to virtual neighborhoods

	Model 1		Model 2		Model 3		Model 4		Model 5		Model 6		Model 7		Model 8		Model 9		Model 10		Model 11		Model 12		Model 13	
	B	*p*	*B*	*p*	*B*	*p*	*B*	*p*	*B*	*p*	*B*	*p*	*B*	*p*	*B*	*p*	*B*	*p*	*B*	*p*	*B*	*p*	*B*	*p*	*B*	*p*
Time	0.05	.028	0.05	.025	0.05	.027	0.05	.024	0.05	.027	0.05	.027	0.05	.025	0.05	.026	0.05	.025	0.05	.025	0.05	.024	0.05	.025	0.05	.03
Condition (Affluent)	-0.04	.95	0.89	.30	0.72	.41	0.95	.28	0.65	.45	0.95	.28	0.92	.30	0.91	.30	0.88	.31	0.86	.33	0.66	.43	0.95	.27	0.59	.52
Parental Education (Below college)			2.43	.026	2.49	.021	2.48	.025	2.14	.048	2.48	.025	2.39	.031	2.41	.029	2.38	.031	2.49	.025	2.42	.022	2.47	.024	2.62	.018
Condition x Parental Education			-2.98	.043	-2.98	.041	-3.15	.039	-2.77	.056	-3.08	.04	-3.04	.042	-3.01	.043	-2.97	.045	-2.99	.044	-2.31	.12	-2.91	.049	-3.08	.036
Age					-0.19	.14																				
Sex (Female = 1)							0.32	.65																		
Education: Graduate student									-1.27	.076																
Video game hours											0.03	.68														
Swiss National													0.21	.79												
Smoking															0.31	.77										
Neighborhood Safety																	-0.24	.73								
Neighborhood Rating																			0.24	.70						
Neighborhood Comparison																					1.06	.033				
Simulator Sickness																							0.00	.45		
City Time																									0.00	.28

Table reports linear mixed models predicting blood pressure reactivity:

- Model 1 examines the main effect of neighborhood condition with no covariates, accounting for the fixed effect of time
- Model 2 examines the interaction between parental education and experimental condition, to test hypotheses concerning sensitization and habituation
- Models 3 – 13 examine the robustness of the interaction between parental education and condition by sequentially testing independence from additional covariates

Table S8. Non-specific skin conductance responses (NS.SCRs) : Response to virtual neighborhoods

	Model 1		Model 2		Model 3		Model 4		Model 5		Model 6		Model 7		Model 8		Model 9		Model 10		Model 11		Model 12		Model 13	
	B	*p*	*B*	*p*	*B*	*p*	*B*	*p*	*B*	*p*	*B*	*p*	*B*	*p*	*B*	*p*	*B*	*p*	*B*	*p*	*B*	*p*	*B*	*p*	*B*	*p*
Time	-0.10	.073	-0.10	.073	-0.10	.073	-0.10	.073	-0.10	.073	-0.10	.073	-0.10	.073	-0.10	.074	-0.10	.073	-0.10	.073	-0.10	.073	-0.10	.073	-0.10	.073
Condition (Affluent)	-0.27	.88	2.73	.21	2.65	.23	2.97	.18	3.08	.16	2.59	.25	2.57	.25	2.74	.22	2.74	.21	2.62	.24	2.67	.23	3.09	.15	2.69	.25
Parental Education (Below college)			4.64	.089	4.65	.090	4.82	.080	5.10	.064	4.53	.10	4.83	.081	4.62	.093	4.78	.084	4.80	.084	4.64	.091	4.84	.072	4.66	.094
Condition x Parental Education			-8.44	.023	-8.44	.024	-9.06	.018	-8.76	.019	-822	.03	-8.14	.031	-8.46	.024	-8.48	.023	-8.43	.024	-8.25	.031	-8.08	.028	-8.45	.024
Age					-0.08	.82																				
Sex (Female)							1.19	.51																		
Education: Graduate student									2.02	.27																
Video game hours											-0.05	.73														
Swiss National													-0.99	.62												
Smoking															0.26	.92										
Neighborhood Safety																	0.79	.66								
Neighborhood Rating																			0.65	.68						
Neighborhood Comparison																					0.32	.81				
Simulator Sickness																							0.004	.11		
City Time																									0.0002	.96

Table reports linear mixed models predicting NS.SCR reactivity:

- Model 1 examines the main effect of neighborhood condition with no covariates, accounting for the fixed effect of time
- Model 2 examines the interaction between parental education and experimental condition, to test hypotheses concerning sensitization and habituation
- Models 3 – 13 examines the robustness of the interaction between parental education and condition by sequentially testing independence from additional covariates
